# The complete mitochondrial genomes of *Diplonevra funebris* and *Diplonevra peregrina* (Diptera: Phoridae)

**DOI:** 10.1080/23802359.2021.1900754

**Published:** 2021-03-18

**Authors:** Dianxing Feng, Dapeng Sun, Shutong Dai

**Affiliations:** College of Life Science and Engineering, Shenyang University, Shenyang, Liaoning, China

**Keywords:** *Diplonevra funebris*, *Diplonevra peregrina*, mtDNA, Phoridae

## Abstract

*Diplonevra* is one of the most important genera in the family Phoridae. This genus is mainly distributed in Palearctic region, and its species can be used to estimate the postmortem interval. In this study, we first present two mitochondrial genomes of common necrophagous species of this genus, *Diplonevra funebris* (Meigen, 1830) and *Diplonevra peregrina* (Wiedemann, 1830). Maximum-likelihood phylogenetic tree revealed that the genus *Diplonevra* is closely related to the genus *Dohrniphora* within the family Phoridae. This work expands the knowledge about the Phoridae genomes, and contributes to the further study of species identification and phylogenetics of this family.

The family Phoridae is usually composed of small individuals (mostly ranging from 1.5–3.0 mm in length). However, one genus of this family, *Diplonevra*, has medium to large specimens (2.0–6.0 mm in length). Besides this difference, the genus *Diplonevra* has 85 known species mostly in the Paleartic region, making it a highly speciose genus (Liu 2001). The species of genus *Diplonevra* are morphologically more similar to those of genus *Dohrniphora* in Phoridae. The genus was also found to relate in vertebrate carrion and was therefore considered to be of forensic importance (Smith 1986; Shalaby et al. 2000; Feng and Liu 2012; Zou et al. 2018). The mitochondrial genome is frequently used for species identification and phylogenetic studies (Fu et al. 2016). In this study, the complete mitogenomes of two common necrophagous flies of this genus, *Diplonevra funebris* (Meigen, 1830) and *Diplonevra peregrina* (Wiedemann, 1830) were reported.

*Diplonevra funebris* (voucher SYU-FD-PHO004) and *D*. *peregrina* (voucher SYU-FD-PHO005) were collected using pork as bait in Shenyang (41°54′5*"*N, 123°24′12*"*E), Liaoning province, China. Each specimen was identified following the taxonomic keys (Liu 2001). The voucher specimens and their DNA were deposited in the Natural History Museum of Shenyang University (https://museum.syu.edu.cn/, Dianxing Feng, fdx0808@163.com). Total DNA was extracted using a DNeasy Blood & Tissue Kit (Qiagen, Hilden, Germany) following manufacturer protocol. The template DNA was 350 bp and the library was constructed with VAHTS^TM^ Universal DNA Library Prep Kit for Illumina® V3 (Vazyme, Nanjing, China), and sequenced using a paired-end strategy 2 × 150 on the Novaseq 6000 platform (Illumina, USA) in Genepioneer Biotechnologies Co. Ltd. (Nanjing, China). The genomes were constructed by mapping the reads against the mitogenomes of Diptera available on the GeneBank database, followed by de novo assembly of the mapped reads using the SPAdes 3.14.1. The mitogenomes were annotated using the MITOS (Bernt et al. 2013) and manually corrected using the NCBI database. The maximum likelihood analysis was performed by RAxML 8.2.10 with 1000 bootstrap replicates (Minh et al. 2013), the optimal evolution model was GTRGAMMA.

The complete mtDNA sequence of *D. funebris* (MW167292) was 15,314 bp long and showed 20.38% of GC. The mtDNA sequence of *D. peregrina* (MW167293) was 15,585 bp long and had 20.39% of GC. Their circular genomes both revealed 13 protein-coding genes (PCG_S_), 22 tRNA genes, 2 rRNA genes and a noncoding Control Region which was 809 bp for *D*. *peregrina* and 456 bp for *D*. *funebris*. Phylogenetic analyses of the five phorid flies with other five species from the Asehiza were performed with two outgroup species, *Ravinia pernix* and *Chrysomya megacephala* from the Schizophora, which indicated the five phorid flies clustered together and formed the family Phoridae (Figure 1). Within the family Phoridae, the genus *Diplonevra* is closely related to the genus *Dohrniphora*.

**Figure 1.**
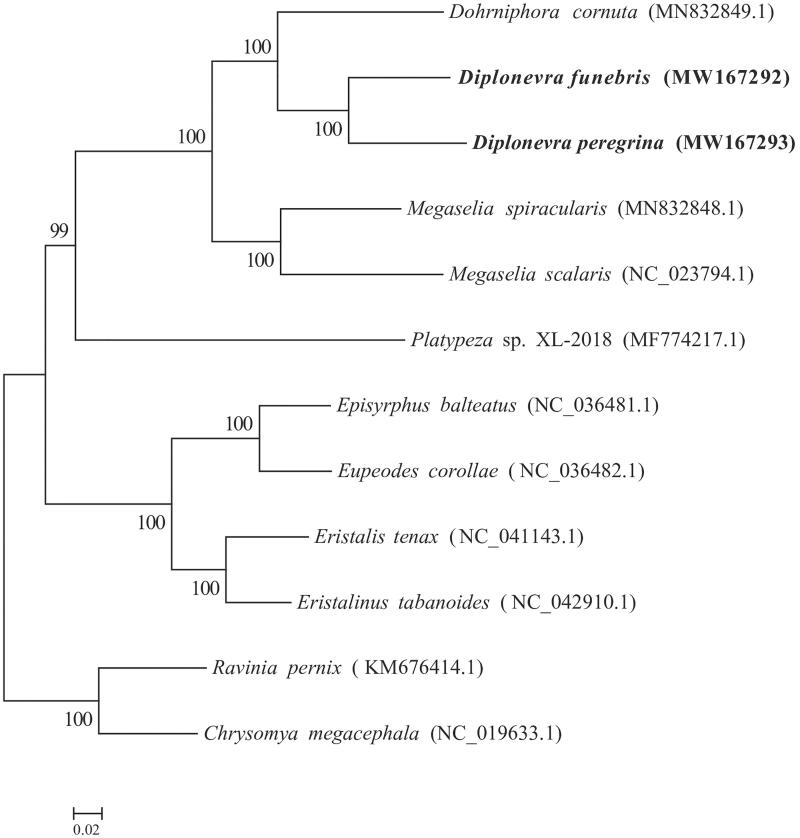
ML phylogenetic tree was constructed based on mitogenome sequences of 10 flies from the Asehiza and two flies from Schizophora as the outgroup using RAxML 8.2.10 software. Number above each node indicates the bootstrap support values with 1000 replicates.

## Data Availability

Mitogenome data supporting this study are openly available in GenBank at nucleotide database, https://www.ncbi.nlm.nih.gov/nuccore/MW167292 and https://www.ncbi.nlm.nih.gov/nuccore/MW167293, Associated BioProject, https://www.ncbi.nlm.nih.gov/bioproject/PRJNA682948, BioSample accession number at https://www.ncbi.nlm.nih.gov/biosample/SAMN17037369 and https://www.ncbi.nlm.nih.gov/biosample/SAMN17014491, Sequence Read Archive at https://www.ncbi.nlm.nih.gov/sra/SRR13224186 and https://www.ncbi.nlm.nih.gov/sra/SRR13228626.
